# Adipocytes promote ovarian cancer chemoresistance

**DOI:** 10.1038/s41598-019-49649-1

**Published:** 2019-09-16

**Authors:** Jiang Yang, Munir M. Zaman, Iliyan Vlasakov, Roopali Roy, Lan Huang, Camilia R. Martin, Steven D. Freedman, Charles N. Serhan, Marsha A. Moses

**Affiliations:** 1 Vascular Biology Program and Department of Surgery, Boston Children’s Hospital, Boston, MA 02115 USA; 2 Department of Surgery, Harvard Medical School, Boston, MA 02115 USA; 3 Division of Gastroenterology, Beth Israel Deaconess Medical Center, Boston, MA 02215 USA; 4 Center for Experimental Therapeutics and Reperfusion Injury, Department of Anesthesiology, Perioperative, and Pain Medicine, Brigham and Women’s Hospital and Harvard Medical School, Boston, MA 02115 USA; 5 Department of Neonatology, Beth Israel Deaconess Medical Center, Boston, MA 02215 USA; 6 Division of Translational Research, Beth Israel Deaconess Medical Center, Boston, MA 02215 USA; 7 Present Address: Department of Comparative Pathobiology, College of Veterinary Medicine, Purdue University, West Lafayette, IN 47906 USA

**Keywords:** Cancer microenvironment, Cancer therapeutic resistance, Chemotherapy, Ovarian cancer

## Abstract

Ovarian cancer (OvCa), while accounting for only 3% of all women’s cancer, is the fifth leading cause of cancer death among women. One of the most significant obstacles to successful OvCa treatment is chemoresistance. The current lack of understanding of the driving mechanisms underlying chemoresistance hinders the development of effective therapeutics against this obstacle. Adipocytes are key components of the OvCa microenvironment and have been shown to be involved in OvCa cell proliferation, however, little is known about their impact on OvCa chemoresistance. In the current study, we found that adipocytes, of both subcutaneous and visceral origin, secrete factors that enhance the resistance of OvCa cells against chemotherapeutic drugs by activating the Akt pathway. Importantly, we have demonstrated that secreted lipids mediate adipocyte-induced chemoresistance. Through a comprehensive lipidomic analysis, we have identified this chemo-protective lipid mediator as arachidonic acid (AA). AA acts on OvCa cells directly, not through its downstream derivatives such as prostaglandins, to activate Akt and inhibit cisplatin-induced apoptosis. Taken together, our study has identified adipocytes and their secreted AA as important mediators of OvCa chemoresistance. Strategies that block the production of AA from adipocytes or block its anti-apoptotic function may potentially inhibit chemoresistance in OvCa patients.

## Introduction

Ovarian cancer (OvCa) is the most lethal cancer of the female reproductive system and the fifth leading cause of cancer death among women (www.cancer.org). One of the most significant obstacles to successful OvCa treatment is chemoresistance. Patients with advanced epithelial OvCa are usually treated with “first line” chemotherapy which is most often a combination of a platinum compound (e.g., cisplatin or carboplatin) and a taxane (e.g., paclitaxel or docetaxel)^1–3^. Although the initial response rate to these drugs is over 80%, the majority of these patients will eventually succumb to resistance and cancer recurrence^4^. These sobering statistics highlight the significant and unmet need for a more thorough understanding of the driving mechanisms underlying chemoresistance and the development of better therapeutics to conquer this clinical challenge.

Ovarian cancer cells reside in an environment rich in adipocytes. Omentum, which is predominantly composed of adipose tissue, is one of the most frequent OvCa metastatic sites. OvCa cells shed into the peritoneal cavity are also in close proximity to other visceral adipose tissues, such as those on the mesenteric membrane and those packed between internal organs such as liver and kidney^5^. Adipocytes in the local environment may promote OvCa progression through production of their own factors. Omental adipocytes have been shown to attract OvCa cells through secreted cytokines such as IL-8 and to transport fatty acids to OvCa cells to support their proliferation^6^. Stroma associated with adipose tissue may also contribute to cancer progression. For example, adipose tissues from obese women are usually infiltrated with macrophages which present a tumor-supporting phenotype^7^. Stromal cells from the adipose tissue of obese individuals have been reported to enhance the proliferation, migration and chemoresistance of OvCa cells^8,9^. Subcutaneous adipose tissues may also exert their effects on OvCa cells over long distances through secreted factors such as adipokines and lipokines^10,11^.

Despite the fact that adipocytes are such key components of the OvCa microenvironment, little is known about their impact on OvCa chemoresistance. In the current study, we found that adipocytes secrete factors that enhance the resistance of OvCa cells against chemotherapeutic drugs by activating the Akt pathway. We have also demonstrated that secreted lipids, not proteins, mediate adipocyte-induced chemoresistance. Finally, we have identified this chemo-protective lipid mediator as arachidonic acid which maybe a potential therapeutic target for combating OvCa chemoresistance. Given the association between obesity, increased risk of OvCa^12–14^ and shorter survival in OvCa patients^15–19^, this study also suggests a mechanistic link between obesity and ovarian cancer.

## Results

### Secreted factors from adipocytes enhance the chemoresistance of ovarian cancer cells

**Figure 1:**
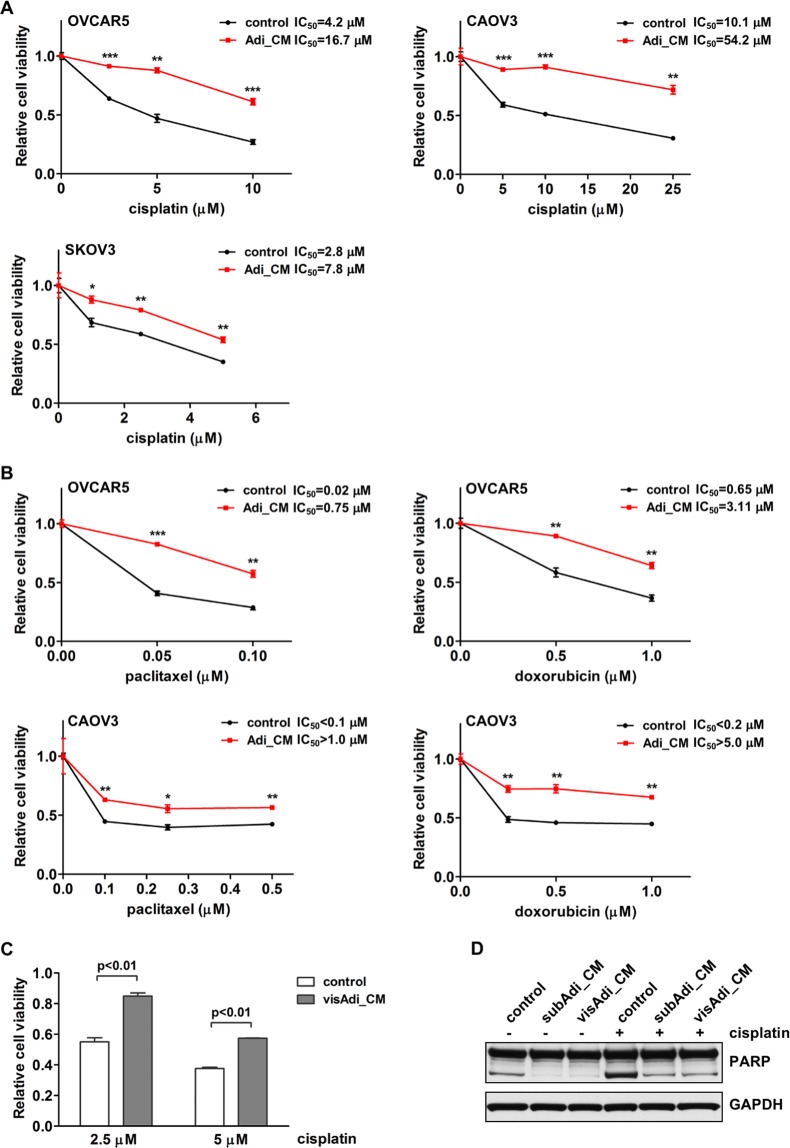
Adipocytes enhance ovarian cancer cell chemoresistance. **(A**) Human OvCa cells OVCAR5, CAOV3 and SKOV3 were incubated with adipocyte CM and different concentrations of cisplatin. Cell viability was analyzed 72 hours later. (**B**) Ovarian cancer cells were incubated with adipocyte CM together with different concentrations of paclitaxel or doxorubicin. (**C**) OVCAR5 cells were incubated with CM from visceral adipocytes (visAdi_CM), together with cisplatin. (**D**) Apoptosis marker cleaved PARP was analyzed in lysates from OVCAR5 cells treated with cisplatin and subcutaneous (subAdi_CM) or visceral adipocyte CM (visAdi_CM). *p < 0.05, **p < 0.01 and ***p < 0.001.

To determine whether adipocytes affect the response of OvCa cells to chemotherapeutic drugs, adipocyte conditioned medium (Adi_CM) was collected from mature human adipocytes. Multiple human OvCa cell lines (OVCAR5, CAOV3 and SKOV3) were treated with Adi_CM together with different concentrations of cisplatin. We found that Adi_CM treatment caused OvCa cells to become more resistant to cisplatin (Fig. 1A). Cell viability in the presence of cisplatin was significantly increased when OvCa cells were treated with Adi_CM. OvCa cells treated with Adi_CM also showed significantly increased resistance to two additional chemotherapeutic drugs commonly used in OvCa treatment, paclitaxel and doxorubicin (Fig. 1B). Significantly increased cell survival in the presence of cisplatin was also observed when cells were treated with CM from human visceral adipocytes (Fig. 1C).

The observed increase in chemoresistance was due to reduced cell apoptosis. Cleaved PARP, an apoptosis marker, was increased in cells treated with cisplatin and its level decreased markedly in OvCa cells treated with either subcutaneous or visceral Adi_CM (Fig. 1D). Both subcutaneous and visceral adipocytes exhibited similar potency against cisplatin-induced apoptosis, suggesting that the capability of adipocytes to promote chemoresistance is independent of the original depot site of the adipocytes. Collectively, these results demonstrate that secreted factor(s) from adipocytes inhibits the apoptosis of OvCa cells induced by chemotherapeutic drugs and therefore promotes their chemoresistance.

### Adipocyte-induced chemoresistance is mediated through the Akt pathway

**Figure 2:**
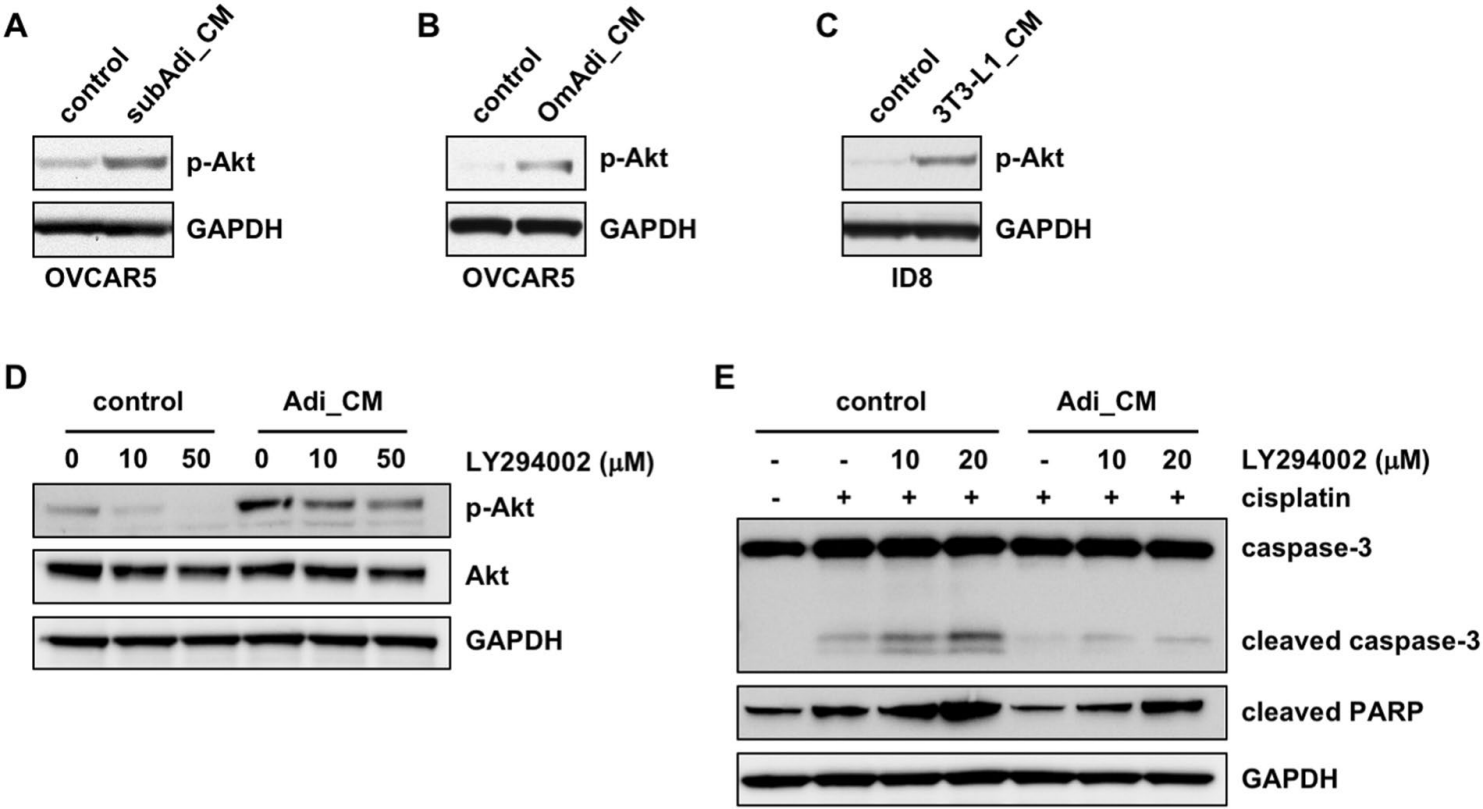
The Akt pathway mediates the anti-apoptotic activity of adipocytes. Akt was activated by both subcutaneous adipocyte CM (subAdi_CM) (**A**) and visceral/omental adipocyte CM (OmAdi_CM) (**B**) in human OVCAR5 cells and by the CM from mouse adipocytes differentiated from 3T3-L1 cells in ID8 mouse OvCa cells (**C**). Adipocyte-induced Akt activation in OVCAR5 cells was inhibited by LY294002 dose-dependently (**D**). LY294002 abolished the anti-apoptotic effect of adipocyte CM, as shown by increased level of cleaved caspase-3 and cleaved PARP (**E**).

We next investigated the signaling pathway that was activated by adipocyte CM in OvCa cells. Given the central role of the PI3K/Akt pathway in cancer cell survival and ovarian cancer chemoresistance, we first looked into this pathway^20,21^. Phosphorylation of Akt at Ser473 has been shown to increase cancer cell survival in the presence of chemotherapeutic drugs^22,23^. Using an antibody specific for Akt phosphorylation at Ser473, we found that Akt phosphorylation was increased in OVCAR5 cells treated with either human subcutaneous or visceral/omental adipocyte CM (Fig. 2A,B). Akt activation was also observed in ID8 mouse OvCa cells treated with CM collected from mouse adipocytes differentiated from 3T3-L1 cells (Fig. 2C). To determine whether Akt activation is critical for the functions of adipocytes, OVCAR5 cells were treated with the Akt pathway inhibitor, LY294002, which effectively inhibited Akt phosphorylation induced by adipocyte CM (Fig. 2D). LY294002 induced apoptosis in cells treated with control media alone, indicating that basal Akt activity is essential for cell survival even under normal circumstances (Supplemental Fig. 1). Consistently, cisplatin caused greater apoptosis in cells treated with LY294002. LY294002-induced cell death was minimal in cells treated with adipocyte CM (Supplemental Fig. 1). Most importantly, LY294002 abolished the anti-apoptotic effect of adipocyte CM and re-induced apoptotic markers such as cleaved caspase-3 and cleaved PARP, in the presence of cisplatin (Fig. 2E), indicating that Akt mediates the anti-apoptotic function of adipocytes. OvCa cells treated with adipocyte CM also showed increased Stat3 activation (Supplemental Fig. 2A), which was most likely induced by IL-6 present in the CM, as blockade of IL-6 in the CM with a neutralizing antibody abrogated Stat3 activation (Supplemental Fig. 2B). Stat3 activation has been shown to contribute to chemoresistance in ovarian cancer^24,25^. However, adipocyte CM, which was treated with the IL-6 neutralizing antibody and was unable to induce Stat3, was still able to block cisplatin-induced apoptosis as effectively as the untreated CM (Supplemental Fig. 2C), suggesting that Stat3 is unlikely to be involved. We did not detect significant activation of Erk and p38 (data not shown) which have been reported to be activated by adipocytes in cancer cells^6^. These results demonstrate that the Akt pathway is critical in the adipocyte-induced chemoresistance in this system.

Adipocytes have been found to function through certain secreted protein factors, the adipokines^10^. Leptin and adiponectin are two major adipokines and both have been shown to activate the Akt pathway^26–28^. To determine whether these two key adipokines are involved in the observed adipocyte-induced chemoresistance, leptin in the adipocyte CM was inhibited with a neutralizing antibody (Supplemental Fig. 3A) and adiponectin production from adipocytes was blocked by transfection with specific siRNAs (Supplemental Fig. 3B). Deprivation of functional leptin or adiponectin had little effect on the ability of adipocyte CM to activate Akt and inhibit cisplatin-induced apoptosis (Supplemental Fig. 3A,C), suggesting that neither of these two adipokines can be implicated in adipocyte-induced chemoresistance.

### Secreted lipid factors mediate adipocyte-induced chemoresistance

**Figure 3:**
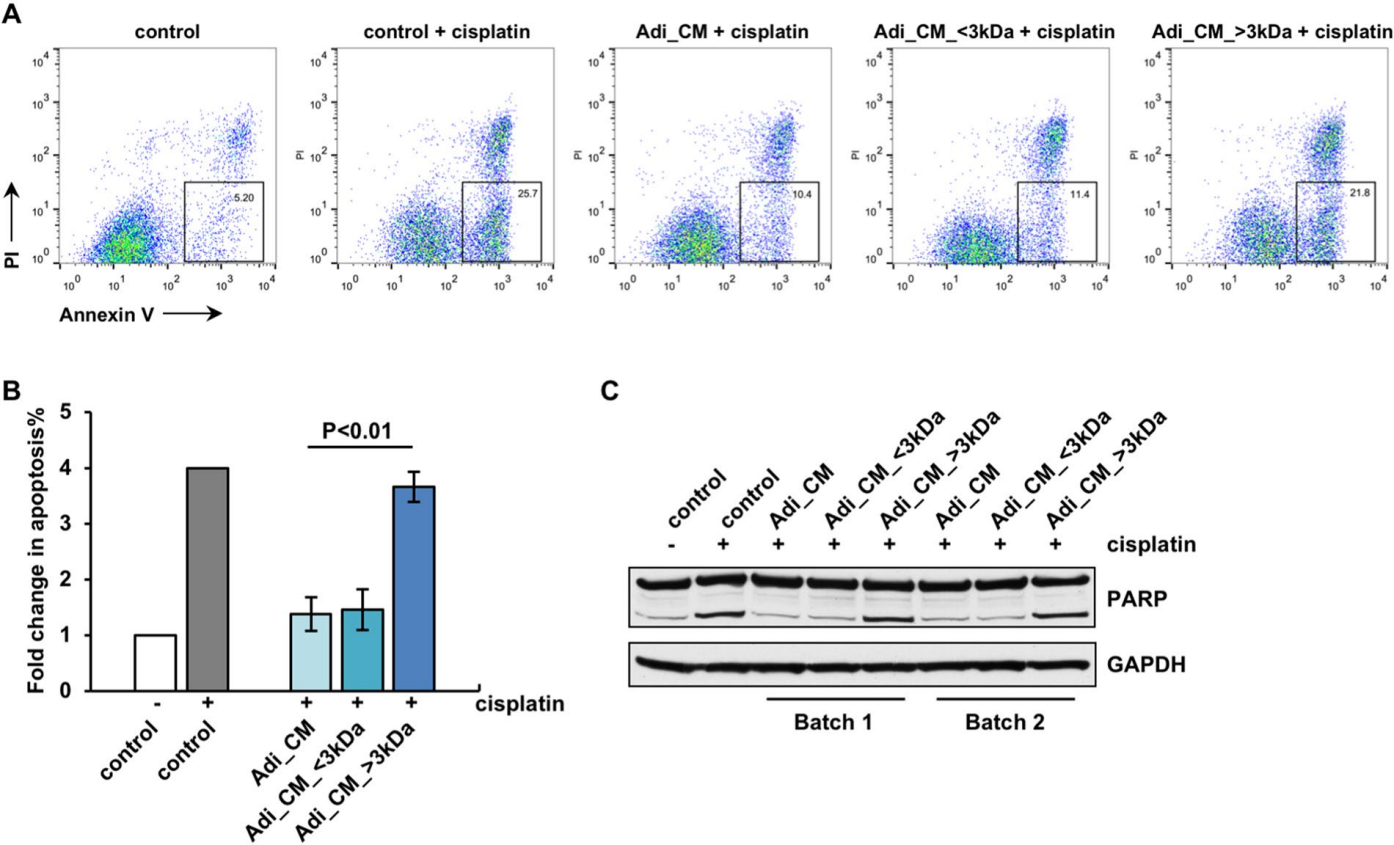
The anti-apoptotic activity of adipocyte CM resides predominantly in the <3 kDa fraction. The <3 kDa fraction of adipocyte CM exerted similar effects as unfractionated adipocyte CM, i.e., reducing the apoptosis in OVCAR5 cells in the presence of cisplatin, whereas the >3 kDa fraction did not. (**A**) Representative dot plot of flow cytometry data after Annexin V staining. (**B**) Fold change in the percentage of Annexin V-positive apoptotic cells in different treatment groups (n = 3). (**C**) PARP immunoblot with two independently collected batches of CM.

To further characterize the factor(s) from adipocytes that enhances OvCa cell chemoresistance, adipocyte CM was separated into <3 kDa and >3 kDa fractions using a centrifugal filter with 3 kDa MWCO (molecular weight cut-off). Interestingly, the <3 kDa fraction, with barely detectable protein levels, exhibited effects similar to that of the unfractionated CM with respect to the anti-apoptosis activity, whereas the >3 kDa fraction which contained ~75% of the total protein in the CM showed little effect. As shown with both Annexin V staining (Fig. 3A,B) and PARP immunoblotting (Fig. 3C), the <3 kDa fraction reduced OvCa cell apoptosis in the presence of cisplatin as effectively as the unfractionated CM whereas the >3 kDa fraction did not.

Given the small molecular weight of the active component(s) in the adipocyte CM, it was unlikely that this active component(s) was a protein. To further rule out this possibility, adipocyte CM was heat-treated at a boiling temperature (95 °C–100 °C) to deactivate protein factors before being applied to OvCa cells with cisplatin. The heat treatment did not alter the ability of adipocyte CM to inhibit cisplatin-induced apoptosis (Supplemental Fig. 4A) or its ability to induce Akt activation (Supplemental Fig. 4B). Moreover, we also separated adipocyte CM into different protein fractions using either sizing (Superdex 200) or anion exchange chromatography (HiTrap Q HP) and none of the protein fractions exhibited an effect on cisplatin-induced apoptosis in ovarian cancer cells (data not shown). These data, together with the negative results obtained with CM deprived of either leptin or adiponectin as described above, strongly indicated that the anti-apoptotic factor(s) in adipocyte CM was unlikely to be a protein(s).

**Figure 4:**
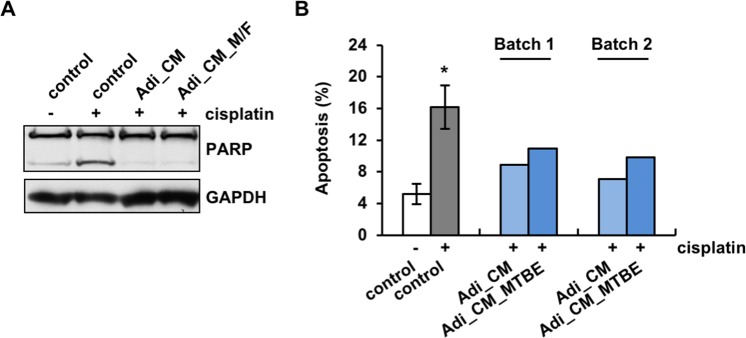
Lipids mediate the anti-apoptotic function of adipocytes. (**A**) Lipids in the <3 kDa fraction of adipocyte CM extracted with methyl formate (M/F) inhibited cisplatin-induced apoptosis in OVCAR5 cells to a similar extent as did the unfractionated CM, as shown by PARP immunoblot. (**B**) Lipids in adipocyte CM extracted with MTBE also reduced cisplatin-induced apoptosis, as shown by Annexin V staining. Two independently collected batches of adipocyte CM (Batch 1 and 2) were analyzed. *p < 0.05.

Given the small molecular weight size of the active component(s) and the unlikeliness of it being a protein, we reasoned that this factor might be a lipid. Lipids secreted from adipocytes have been shown to modulate the behavior of other cell types^29^. In order to test this hypothesis, we extracted lipids from the <3 kDa fraction^30,31^ using a C18 hydrophobic column eluted with methyl formate (M/F) and tested them on OVCAR5 cells together with cisplatin. The lipids in the eluted fraction markedly reduced cisplatin-induced apoptosis as effectively as unfractionated CM (Fig. 4A), suggesting that lipids account for the majority of the anti-apoptotic activity of adipocytes. The critical function of lipids in chemoresistance in these studies was further corroborated with lipids extracted from whole adipocyte CM using methyl-tert-butyl ether (MTBE)^29,32^. Lipids extracted with MTBE were also shown to decrease the apoptotic population after cisplatin treatment to an extent comparable to the adipocyte CM (Fig. 4B).

### Anti-apoptotic lipid factor(s) identified through lipidomic analysis

To identify the lipid mediators secreted from adipocytes that exert the observed anti-apoptotic effect, a lipidomic analysis was conducted on the lipid fraction of adipocyte CM. Arachidonic acid (AA, omega-6 polyunsaturated fatty acid, PUFA), eicosapentaenioc acid (EPA, omega-3 PUFA) and docosahexaenoic acid (DHA, omega-3 PUFA) were identified at 43.475 ± 9.011 pg/mL, 25.325 ± 7.899 pg/mL and 35.075 ± 5.977 pg/mL, respectively (Table 1). Interestingly, lipid mediators derived from AA, such as prostaglandins (PGD_2_, PGE_2_ and PGF_2α_) and lipoxins (LXA_4_ and AT-LXA_4_) were present in adipocyte CM, whereas lipid mediators derived from EPA or DHA, such as D- or E-series resolvins (RvDs and RvEs), protectin D1 (PD1) and maresin 1 (Mar1), were not (Table 1).

**Table 1:** Lipidomic analyses of the <3 kDa fraction of adipocyte-conditioned media.

AA bioactive metabolome	Conditioned media extracts (pg/mL) (n = 4)
AA	43.5 ± 9.0
PGD_2_	1.8 ± 0.4
PGE_2_	5.6 ± 2.9
PGF_2α_	4.0 ± 0.7
LXA_4_	6.6 ± 1.7
AT-LXA_4_	31.2 ± 4.4
Below limit (<0.1 pg)	LXB_4_, 5S, 15S-diHETE, AT-LXB_4_, LTB_4_, 20-OH-LTB_4_, 20-COOH-LTB_4_, 5S,12S-diHETE, TXB_2_
**EPA bioactive metabolome**	**Conditioned media extracts (pg/mL) (n** = **4)**
EPA	25.3 ± 7.9
Below limit (<0.1 pg)	RvE1, RvE2, RvE3
**DHA bioactive metabolome**	**Conditioned media extracts (pg/mL) (n** = **4)**
DHA	35.1 ± 6.0
Below limit (<0.1 pg)	RvD1, RvD2, RvD3, RvD4, RvD5, RvD6, AT-RvD1, AT-RvD3, PD1, AT-PD1, 10S,17S-diHDHA, 22-OH-PD1, Mar1, 7S, 14S-diHDHA, 4S, 14S-diHDHA

**Figure 5:**
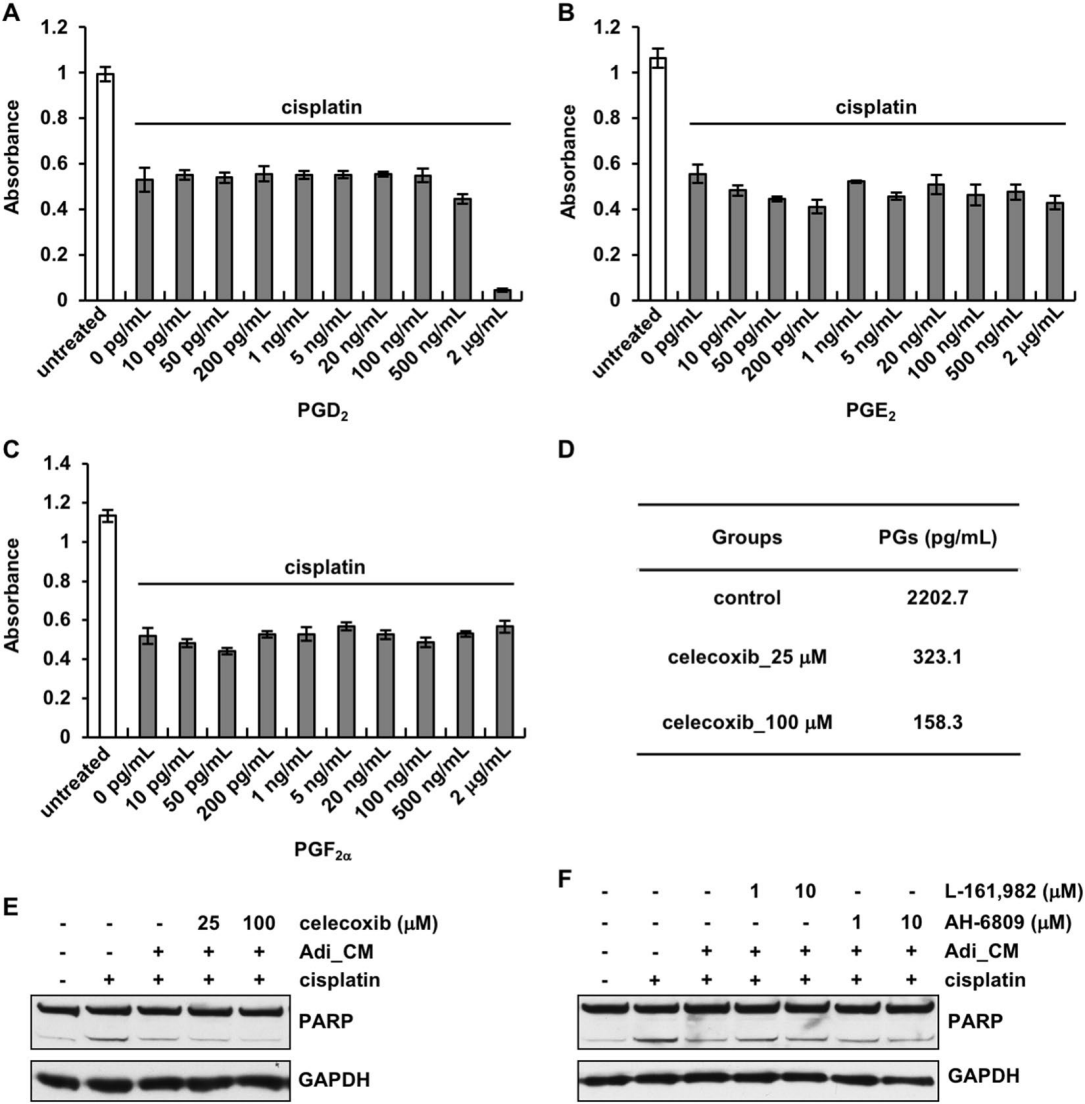
Prostaglandins do not mediate adipocyte-induced chemoresistance. Prostaglandin D_2_ (PGD_2_) (**A**), PGE_2_ (**B**) and PGF_2α_ (**C**) at different concentrations were applied on OVCAR5 cells in the presence of 1 μM cisplatin. Cell viability was analyzed after 72 hours. PGD_2_ applied at 2 μg/mL caused extensive cytotoxicity. (**D**) Prostaglandin levels were analyzed in the CM collected from adipocytes treated with COX1 and COX2 inhibitor celecoxib. (**E**) Blockade of prostaglandin synthesis with celecoxib did not compromise the anti-apoptotic function of adipocytes. (**F**) Adipocyte CM treatment reduced cisplatin-induced apoptosis even when PGE_2_ receptors were blocked on OVCAR5 cells with specific inhibitors.

The lipids identified from the lipidomic analysis were then tested individually on OvCa cells in the presence of cisplatin. The identified prostaglandins (PGD_2_, PGE_2_ and PGF_2α_) showed little effect against cisplatin. Cell viability was reduced by cisplatin to the same extent with or without the presence of different concentrations of PGD_2_, PGE_2_ or PGF_2α_ (Fig. 5A–C). Whether prostaglandins contribute to adipocyte-induced chemoresistance was further investigated by treating adipocytes with celecoxib (cyclooxygenase (COX) 1 and 2 inhibitor) to block the synthesis of prostaglandins. Prostaglandin levels secreted from adipocytes, as analyzed with a prostaglandin screening ELISA, were reduced in a dose-dependent manner after celecoxib treatment (Fig. 5D). However, depletion of prostaglandins did not diminish the anti-apoptotic effect of the adipocyte CM (Fig. 5E). Given that PGE_2_ has been implicated in chemoresistance^33,34^, OvCa cells were also treated with PGE_2_ receptor antagonists (L-161,982 and AH-6809) to block the function of PGE_2_. Consistent with the results from COX inhibition experiments, blockade of the PGE_2_ receptors on OvCa cells did not prevent adipocyte CM from protecting against cisplatin-induced apoptosis (Fig. 5F). Taken together, these results indicate that prostaglandins, although present in the adipocyte CM, are not involved in adipocyte-induced ovarian cancer chemoresistance.

Our data have also shown that lipoxins, another major lipid population identified in adipocyte CM through lipidomics, are not implicated with respect to OvCa chemoresistance. OvCa cells treated with different concentrations of lipoxin A_4_ (LXA_4_) or AT-LXA_4_ did not exhibit any changes in their sensitivity towards cisplatin (Supplemental Fig. 5A). Furthermore, CM from adipocytes treated with lipoxygenase (LOX) inhibitors to block lipoxin synthesis was only mildly changed in potency compared to CM from untreated adipocytes (Supplemental Fig. 5B).

### Arachidonic acid is an active component secreted from adipocytes that promotes ovarian cancer chemoresistance

**Figure 6:**
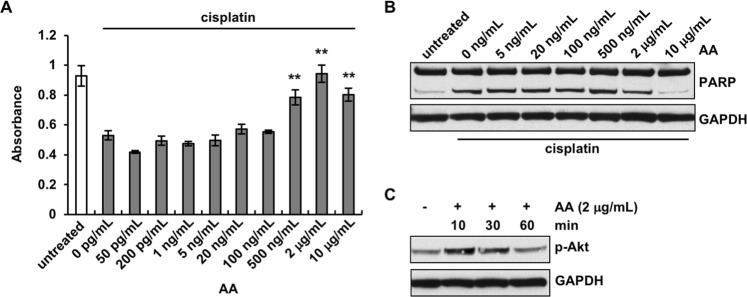
Arachidonic acid induces chemoresistance in ovarian cancer cells. **(A**) OVCAR5 cells were treated with 1 μM cisplatin and different concentrations of arachidonic acid (AA). Cell viability was analyzed 72 hours later. (**B**) AA inhibited cisplatin-induced apoptosis, as demonstrated by PARP immunoblotting. (**C**) Akt was activated in OVCAR5 cells by AA. **p < 0.01, as compared to control cells treated with cisplatin.

After determining that none of the downstream derivatives of AA were involved in ovarian cancer chemoresistance, we next asked whether AA itself might directly act on OvCa cells. Significantly increased cell viability in the presence of cisplatin was observed when cells were treated with AA ranging from 500 ng/mL to 10 μg/mL, in a dose-dependent fashion, with the most significant effect observed with 2 μg/mL of AA (Fig. 6A). Cisplatin-induced cytotoxicity was actually completely abrogated when OvCa cells were treated with 2 μg/mL AA. Consistently, reduced levels of cleaved PARP in the presence of cisplatin were observed in cells treated with AA at 2 or 10 μg/mL, but not with AA at lower concentrations (Fig. 6B). Importantly, AA was found to activate Akt in OvCa cells (Fig. 6C). Akt phosphorylation was detected in OvCa cells as early as 10 min after the addition of AA. Similarly, adipocyte CM and AA also significantly reduced the cytotoxicity induced by carboplatin, another platinum-based compound commonly used in ovarian cancer chemotherapy (Supplemental Fig. 6). Taken together, these data demonstrate that AA is capable of activating Akt and inhibiting cisplatin-induced apoptosis.

**Figure 7:**
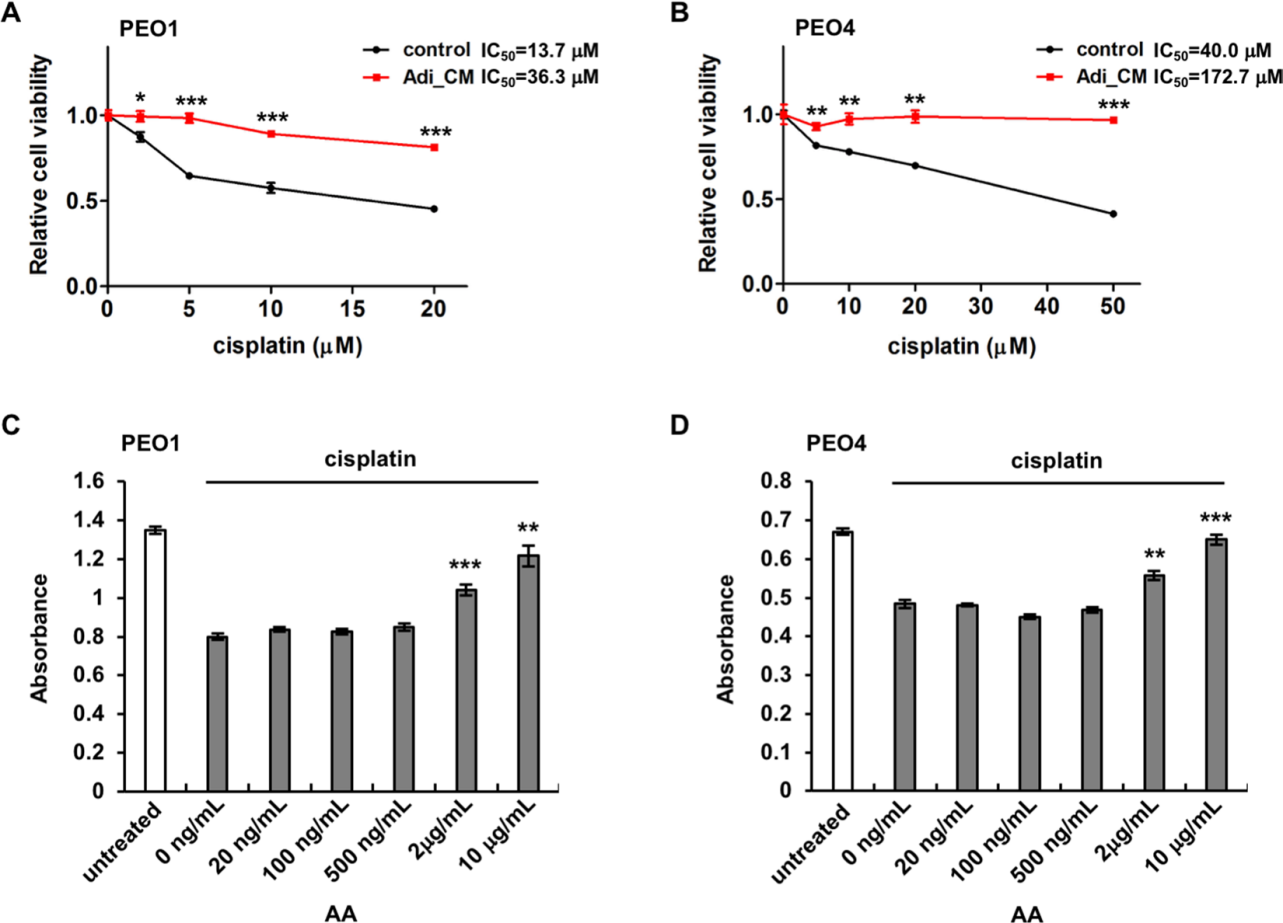
Adipocyte CM and arachidonic acid increase chemoresistance in both chemo-sensitive and -resistant ovarian cancer cells. Chemosensitve PEO1 cells (**A**) and chemoresistant PEO4 cells (**B**) were incubated with adipocyte CM and different concentrations of cisplatin. PEO1 cells (**C**) and PEO4 cells (**D**) were also treated with 5 μM or 20 μM cisplatin, respectively, and different concentrations of arachidonic acid (AA). Cell viability was analyzed 72 hours later. *p < 0.05, **p < 0.01 and ***p < 0.001.

We have also evaluated the effects of adipocyte CM and AA on both chemo-sensitive and -resistant OvCa cells. PEO1 and PEO4 cells were derived from the same patient before and after the development of resistance to cisplatin^35,36^. The two lines displayed different sensitivity to cisplatin as expected, with approximately 40% PEO1 cells surviving with 20 μM cisplatin and approximately 40% PEO4 cells surviving even with 50 μM cisplatin (Fig. 7A,B). We have found that adipocyte CM enhanced the survival of both PEO1 and PEO4 in the presence of cisplatin, regardless of their sensitivity (Fig. 7A,B). Strikingly, 97% of PEO4 cells survived with the protection of adipocyte CM even at the highest cisplatin dose tested (50 μM), whereas only 41% survived with the control media at the same cisplatin dosage. Consistent with AA being the effective component in adipocyte CM in terms of promoting chemoresistance, AA significantly enhanced the survival of both lines, dose-dependently, when they were treated with 5 or 20 μM cisplatin, respectively (Fig. 7C,D). These data demonstrate that adipocytes and their secreted AA provide a general survival advantage to OvCa cells and render them more chemoresistant, regardless of their intrinsic sensitivity to chemotherapeutic drugs. Consistently, cell survival has been identified as one of the most activated pathways by adipocytes in OvCa cells^37^.

## Discussion

The omentum, which is composed predominantly of adipose tissue, is one of the most frequent metastatic sites for ovarian cancer. As a result, studies of the interactions between adipocytes and OvCa cells have largely focused on the question as to why the omentum is the preferred dissemination site for ovarian cancer. It has been shown that omental adipocytes release chemoattractant cytokines, such as IL-8, to stimulate the migration of OvCa cells towards omentum^6^. These omental adipocytes also produce fatty acids, which can be used as an energy source, to sustain the rapid growth of OvCa cells that settle at the site. The importance of adipocytes in the establishment of omental metastasis has been further corroborated by the finding that adipocytes activate salt-inducible kinase 2 in ovarian cancer cells and stimulate fatty acid metabolism^38^. However, adipocytes may be responsible for other elements of ovarian cancer progression than the preparation of a metastatic niche alone. Obesity has been significantly associated with a worse outcome for ovarian cancer patients^15–19^, suggesting that adipose tissue may not only support the proliferation of ovarian cancer cells but also protect the cancer cells from the cytotoxicity of chemotherapeutic drugs, causing the cancer to be more resistant and more likely to recur once the therapy stops.

Our current study has demonstrated that adipocytes can indeed enhance the chemoresistance of OvCa cells by reducing their apoptosis in the presence of chemotherapeutic drugs. More importantly, we have identified an important mechanism underlying adipocytes-mediated chemoresistance. We have found that adipocytes exert their chemoresistant function through a secreted factor which activates the Akt pathway in OvCa cells and inhibits the apoptosis induced by chemotherapeutic drugs. Combined with the observation that both visceral/omental and subcutaneous adipocytes increase OvCa cell chemoresistance to a similar extent, this adipocyte-derived factor may act remotely and systemically on OvCa cells. Another significant implication of our findings is the fact that this factor can be detected in the circulation and may therefore serve as a blood or urinary biomarker to identify patients who are likely to develop chemoresistance in order that additional or alternative therapies can be provided to these patients in a timely fashion to improve their outcome.

By performing a series of fractionation and characterization studies, we have, for the first time, identified the adipocyte-secreted chemo-protective factor(s) as a lipid(s). Although a recent study has shown that adipocytes derived from mouse NIH 3T3-L1 fibroblasts, not human cells, also enhance ovarian cancer cell chemoresistance^39^, the active chemo-protective factor(s) from adipocytes was not identified. Through our lipidomic analyses, omega-6 PUFA (AA) and omega-3 PUFAs (EPA and DHA), as well as downstream derivatives from AA, such as several prostaglandins and lipoxins, have been identified as being secreted from adipocytes. Interestingly, resolvins, protectin D1 and maresin 1, which are derived from EPA and DHA, were not detected in this study. Prostaglandins have been found to play active roles in cancer chemoresistance. For example, PGE_2_ has been shown to induce the repopulation of bladder cancer stem cells which are responsible for cancer recurrence after chemotherapy^34^. PGE_2_ has also been reported to contribute to pancreatic cancer chemoresistance^33^. We have studied the functions of PGE_2_ by using purified PGE_2_, interrupting its function by blocking its synthesis in adipocytes and antagonizing its receptors on OvCa cells. None of these studies provided any evidence that PGE_2_ is involved in the adipocyte-induced chemoresistance observed in our study. Other identified prostaglandins, such as PGD_2_ and PGF_2α_, as well as lipoxins have also been ruled out as the active agents using similar experiments in this study.

We postulate that arachidonic acid, and not its downstream derivatives, may act directly on OvCa cells to induce chemoresistance. We have shown that AA completely inhibited the cytotoxicity of cisplatin. It increased the viability of OvCa cells in the presence of cisplatin, to the same level as that of untreated cells. Our data has also demonstrated that AA induces Akt activation in OvCa cells. The level of Akt activation positively correlated with the chemo-protective effect of AA. The mechanisms underlying AA’s actions on OvCa cells await further investigation. AA could be incorporated into the lipid layer of the cell membrane and modulate the configuration of lipid rafts, as have shown in neural cells and immune cells^40–42^. Consequently, AA may alter the organization, distribution and activity of cell surface proteins that are involved in drug resistance such as Pgp and MRP1 localized in the lipid rafts^43^. AA might also be processed within OvCa cells into downstream derivatives which further induce chemoresistance. Consistent with this possibility, increased COX and LOX activity has been described in ovarian cancer^44–46^. Therefore, strategies that block AA synthesis or secretion from adipocytes or block its anti-apoptotic function on OvCa cells may potentially inhibit chemoresistance and lead to novel therapeutics against ovarian cancer and enhance the survival of ovarian cancer patients.

## Materials and Methods

### Reagents

Cisplatin, carboplatin, paclitaxel, doxorubicin, and celecoxib were purchased from Sigma-Aldrich (St. Louis, MO). AA, PGD_2_, PGE_2_, PGF_2α_, LXA_4_, AT-LXA_4_, L-161,982 and AH-6809 were purchased from Cayman Chemical (Ann Arbor, MI). IL-6 and leptin neutralizing antibodies, IgG controls, LY294002, MK886, BW-B 70C and PD 146176 were purchased from R&D Systems (Minneapolis, MN).

### Cell culture

OVCAR5 cells were obtained from the Tissue Culture Core of the Vascular Biology Program at Boston Children’s Hospital and cultured in Dulbecco’s modified Eagle’s medium (DMEM; Thermo Fisher Scientific, Waltham, MA) supplemented with 10% fetal bovine serum (FBS, Atlanta Biologicals, Flowery Branch, GA). CAOV3 and SKOV3 were purchased from American Type Culture Collection (ATCC; Manassas, VA) and cultured in DMEM (Thermo Fisher) and McCoy’s 5 A medium (ATCC), respectively, supplemented with 10% FBS. ID8 cells were kindly provided by Dr. Jack Lawler at Beth Israel Deaconess Medical Center and cultured in DMEM supplemented with 2% FBS. PEO1 and PEO4 cells were purchased from Sigma-Aldrich and cultured according to the protocols provided.

### Adipocyte culture and treatment

Human subcutaneous preadipocytes were purchased from Cell Applications Inc. (San Diego, CA). Human visceral/omental preadipocytes and mouse 3T3-L1 preadipocytes were purchased from Zen-Bio Inc. (Research Triangle Park, NC). These cells were maintained and differentiated into mature adipocytes as instructed by the suppliers.

To prepare the conditioned medium (CM), mature adipocytes were cultured in serum-free Adipocyte Basal Medium (Cell Applications) for 24 h. Supernatant was cleared of cell debris through centrifugation and collected as CM. Adipocyte Basal Media was used as control for CM in all relevant experiments.

To block COX or LOX activity, mature adipocytes were treated with the COX inhibitor (celecoxib) or LOX inhibitors (MK 886, BW-B 70 C and PD 146176) for 24 hours before CM was collected. IL-6 or leptin activity in adipocyte CM was blocked by pre-treating CM with anti-IL-6 or anti-leptin neutralizing antibodies for one hour at 37 °C before the CM was applied to OvCa cells. In order to inhibit adiponectin production, mature adipocytes were transfected with a siRNA pool against adiponectin (GE Dharmacon, Lafayette, CO) or control non-targeting siRNAs using Lipofectamine RNAiMAX Reagent (Thermo Fisher) according to manufacturer’s instructions.

### Cell viability assays

OvCa cells were seeded in 96-well plates (OVCAR5 at 4,000 cells/well, PEO1 and PEO4 at 10,000 cells/well) the day before treatments with chemotherapeutic drugs, adipocyte CM (supplemented with 1% FBS) or lipids as described in the main text. Cell viability was analyzed 72 hours later using Cell Counting Kit-8 (Donjindo Molecular Technologies, Rockville, MD) according to manufacturer’s instructions. The assay was repeated independently 2 or 3 times with 3–4 replicates in each experiment.

### Annexin V staining

Floating cells were pelleted together with adherent cells that were detached using Accutase (Stemcell Technologies, Vancouver, Canada) 24 hours after treatment. Cell pellets were washed three times with cold phosphate-buffered saline (PBS). Cell staining was performed using Alexa Fluor 488 Annexin V/Dead Cell Apoptosis Kit (Thermo Fisher) according to manufacturer’s instructions. Stained cells were analyzed by flow cytometry on BD FACSCalibur (Becton Dickinson, Franklin Lakes, NJ). Data were analyzed using FlowJo software (FlowJo, LLC, Ashland, OR).

### Immunoblot analyses

Whole-cell lysates were collected using RIPA lysis buffer (Santa Cruz Biotechnology, Santa Cruz, CA). For apoptosis marker immunoblots, lysates from both the floating and adherent cells were collected 24 hours after treatment. For the experiments with LY294002, cells were pre-treated with LY294002 for one hour at indicated concentrations and then treated with adipocyte CM and cisplatin together with LY294002. For Akt activation, cell lysates were collected 30 minutes after adipocyte CM treatment. Proteins were separated on NuPAGE 4–12% Bis-Tris gels (Thermo Fisher) and transferred to nitrocellulose membrane (Bio-Rad, Hercules, CA). The membrane was blocked in TBST (Tris-buffered saline with 0.2% Tween-20) with 5% milk for 1 hour and incubated with primary antibodies diluted (1:1000) in TBST with 2% milk overnight at 4 °C and secondary antibodies (Thermo Fisher) (diluted 1:10,000) for 1 hour at room temperature. The membrane was developed using Western Lightning Plus-ECL Enhanced Chemiluminescence Substrate (PerkinElmer, Waltham, MA). The following antibodies were used: anti-PARP, anti-cleaved PARP, anti-caspase-3, anti-cleaved caspase-3, anti-phospho-Akt (Ser473), anti-Akt, anti-phospho-Stat3, anti-Stat3 (Cell Signaling Technology, Danvers, MA), and anti-glyceraldehyde-3-phosphate dehydrogenase (GAPDH; MilliporeSigma, Billerica, MA).

### Lipid extraction

Adipocyte CM was first separated into <3 kDa and >3 kDa fractions using Amicon Ultra-15 Centrifugal Filter Units (3 kDa MWCO, MilliporeSigma) and the <3 kDa fraction was subsequently used for lipid extraction using methyl formate as described^31^. Briefly, equal volume of methanol was added to CM and the mixture was incubated at −20 °C for 30 min to allow protein precipitation and then centrifuged at 4 °C at 1,200 *g* for 10 min. Supernatants were collected and the methanol content was brought to less than 10% of total volume under a gentle stream of nitrogen gas. Nine mL H_2_O (pH 3.5, HCl) were added and the acidified solutions were rapidly loaded onto conditioned Biotage Isolute C18 cartridges (Biotage, Uppsala, Sweden) that were washed with 6 mL H_2_O. Next, 7 mL hexane were added and products eluted with 7 mL methyl formate. Products were brought to dryness using a gentle stream of nitrogen gas and immediately suspended in methanol-water (50:50 vol/vol) for the liquid chromatography-tandem mass spectrometry system (LC-MS-MS) automated injections or suspended in Adipocyte Basal Media (Cell Applications) supplemented with 2% fatty acid-free bovine serum albumin (BSA) (Sigma-Aldrich) for cell treatment.

Lipids in CM were also extracted using methyl-tert-butyl-ether (MTBE) as described previously^29^. Briefly, 5 mL MTBE was added to 1 mL CM. The mixture was vortexed for 30 min at room temperature and centrifuged at 1,000 *g* for 10 min. The lipid-containing upper layer was collected. The extraction procedure was repeated. The combined lipid-containing fraction was then dried in a gentle stream of nitrogen gas and reconstituted to 1 mL with Adipocyte Basal Media (Cell Applications) supplemented with 2% fatty acid-free BSA (Sigma-Aldrich).

### Lipidomic analyses

LC-MS-MS data acquisition and analyses were performed as described previously^31,47^. Briefly, to facilitate lipid mediator quantitation and sample recovery, 5 deuterium labeled internal standards were added to each sample: d8-5-HETE, d5-RvD2, d5-LXA_4_, d4-LTB_4_, d4-PGE_2_ (500 pg each; Cayman). Samples were then injected into the LC-MS-MS system, which consisted of a Qtrap 5500 (Sciex, Framingham, MA) equipped with a Shimadzu LC-20AD HPLC (Tokyo, Japan) and data was acquired with parameters described previously^31,47^. Targeted MRM (multiple reaction monitoring) was utilized to quantify the lipid mediator levels. Lipid mediators below the limit of detection (~0.1 pg) were deemed to be non-detectable. Data analysis was performed on the Sciex software platform (Analyst version 1.6).

### Protein fractionation

All column chromatography experiments were performed using protocols described previously^48,49^. Columns included the sizing column Superdex 200 (10/300, bed volume 24 mL, GE Healthcare, Pittsburgh, PA) and the anion exchange column HiTrap Q HP (bed volume 5 mL, GE) run on a LCC 500 FPLC system (GE) attached to a detector (Pharmacia LKB, optical unit UV-1). For protein fractionation based on molecular weight, a Superdex 200 gel filtration column was first equilibrated with 3–5 column volumes (CV) of equilibration buffer (50 mM Tris, 50 mM NaCl). Adipocyte CM (30 mL) was concentrated using Amicon Ultra-15 (3 kDa MWCO, MilliporeSigma) and 0.5 mL of the concentrate was loaded into the column. Fractions were eluted at the rate of 0.5 mL/min and collected. A second fractionation strategy was based on ion exchange chromatography using a HiTrap Q HP column. Adipocyte CM was loaded onto the column at a rate of 1 mL/min and the column was washed with 5–10 CV of equilibration buffer (50 mM Tris, 50 mM NaCl). Fractions were then eluted using a stepwise gradient of buffer (50 mM Tris containing 100–500 mM of NaCl). For all purification steps, fractions collected were buffer-exchanged into Adipocyte Basal Medium (Cell Applications) and concentrated using Amicon Ultra-15 (3 kDa MWCO).

### Prostaglandin ELISA

Prostaglandins in Adipocyte CM were analyzed using the Prostaglandin Screening ELISA Kit (Cayman) according to protocols provided by the manufacturer.

### Real Time-PCR (RT-PCR)

RNA was collected using the RNeasy Kit (Qiagen, Valencia, CA) and then treated with DNase I (Thermo Fisher) before the cDNA was synthesized using random primers and SuperScript III reverse transcriptase (Thermo Fisher). Real-time PCR was performed using the PerfeCTa SYBR Green SuperMix (Quanta Biosciences, Gaithersburg, MD). All of the above procedures were performed according to manufacturer’s instructions. The primers used for PCR were: adiponectin, forward, 5′-TGGTCCTAAGGGAGACATCG-3′, reverse, 5′-CAATCCCACACTGAATGCTG-3′; GAPDH, forward, 5′-AGCCACATCGCTCAGACAC-3′, reverse, 5′-AATGAAGGGGTCATTGATGG-3′.

### Statistical analyses

The values from 2 groups were compared using Student’s *t* test, and values from multiple groups were compared using 1-way ANOVA (GraphPad Prism, La Jolla, CA). Values of *p* < 0.05 were considered statistically significant. Data are presented as means ± SEM.

## Supplementary information

Supplemental Figures

## Data Availability

All data generated or analyzed during this study are included in the manuscript (and its Supplementary Information files).
