# Supplementary material for: Adipocytes promote ovarian cancer chemoresistance
Source: Sci Rep. 2019 Sep 16;9:13316. doi: 10.1038/s41598-019-49649-1 (PMC6746782; doi:10.1038/s41598-019-49649-1)
Supplement: Supplementary file 1 — Supplemental Figures [file 41598_2019_49649_MOESM1_ESM.pdf]

Adipocytes promote ovarian cancer chemoresistance

Jiang Yang, Munir M. Zaman, Iliyan Vlasakov, Roopali Roy, Lan Huang, Camilia R. Martin, Steven D. Freedman, Charles N. Serhan and Marsha A. Moses

Supplemental Figure 1

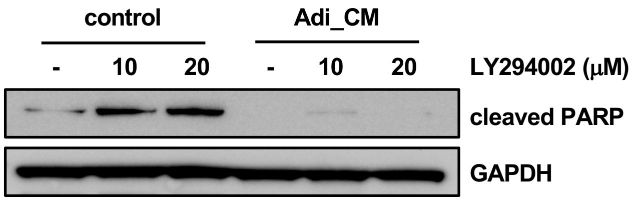

Supplemental Figure 1. LY294002 induces minimal cell death in OVCAR5 cells treated with adipocyte CM.

Supplemental Figure 2

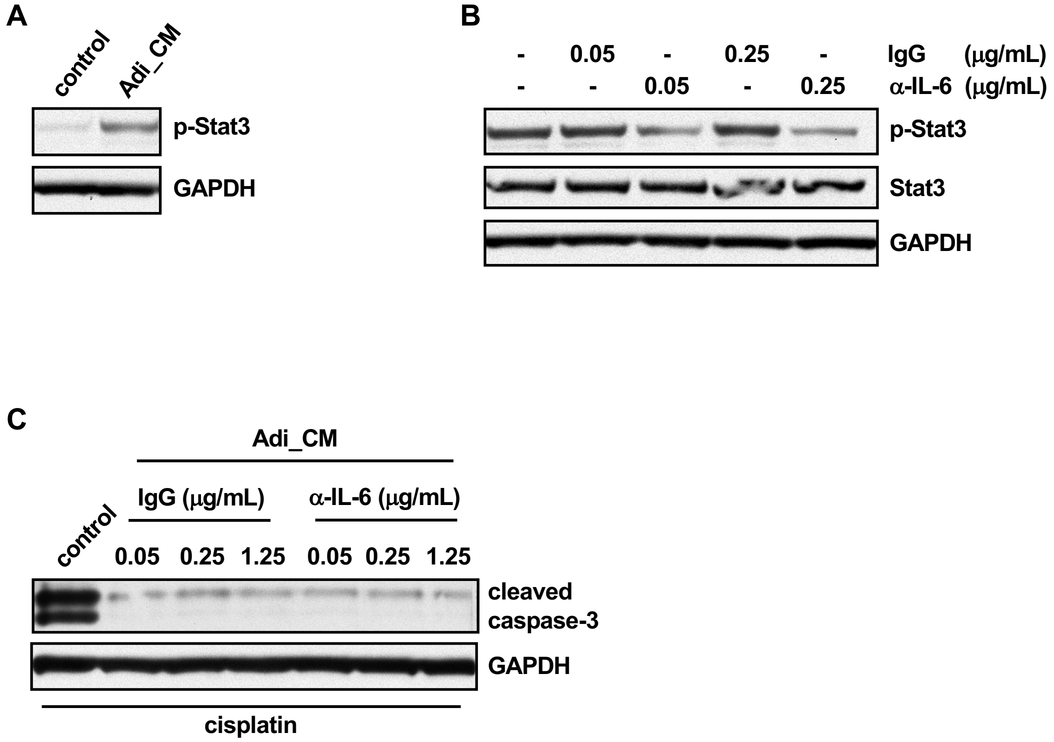

Supplemental Figure 2. Adipocyte-induced chemoresistance is not mediated through the IL-6/Stat3 pathway. A) Adipocyte CM induced Stat-3 phosphorylation. **B**) Treatment of adipocyte CM with IL-6 neutralizing antibody inhibited Stat3 phosphorylation. **C**) Cisplatin-induced apoptosis was inhibited in the presence of adipocyte CM; depletion of IL-6 from the CM did not diminish the chemo-protective effects.

Supplemental Figure 3

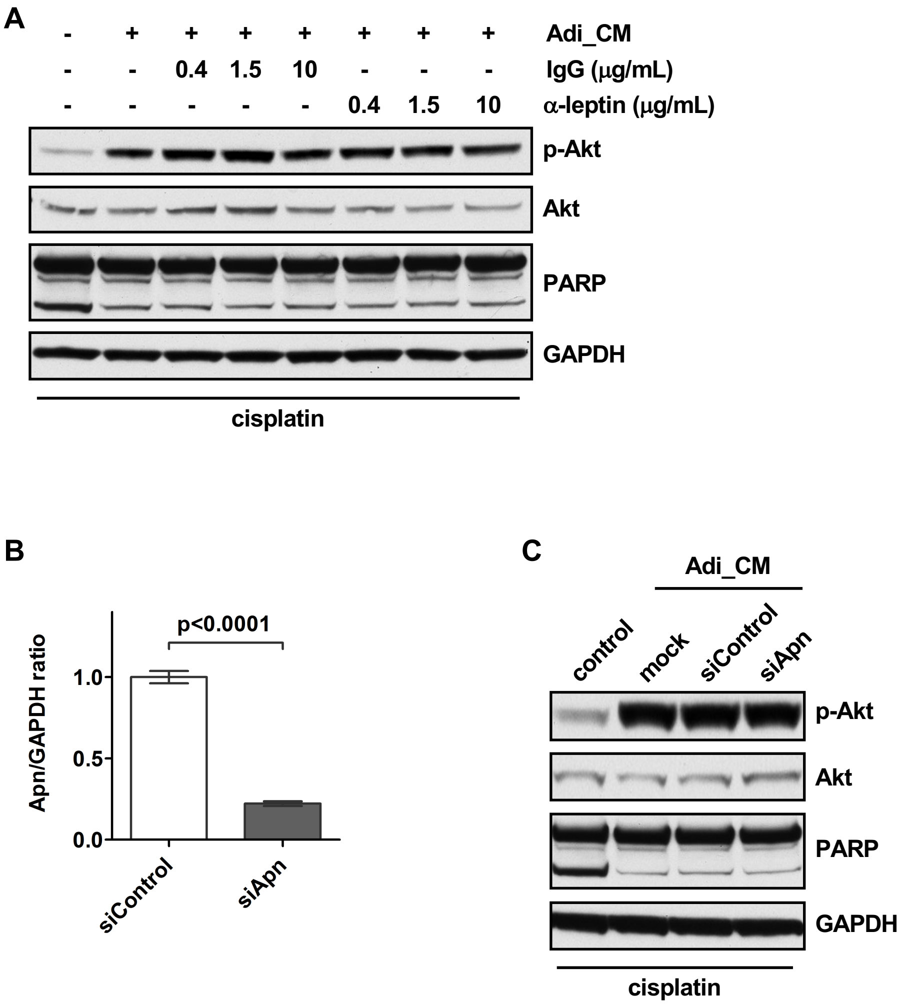

Supplemental Figure 3. Adipocyte-induced chemoresistance is not mediated through leptin or adiponectin. A) Leptin was blocked in the adipocyte CM with a neutralizing antibody and the lack of functional leptin did not compromise the effects of CM on Akt activation and cisplatin-induced apoptosis. **B**) Adiponectin (Apn) was silenced in adipocytes with specific siRNAs and its levels were analyzed with RT-PCR. **C**) Removal of adiponectin in adipocyte CM did not affect CM-induced Akt activation and CM-induced chemoresistance.

Supplemental Figure 4

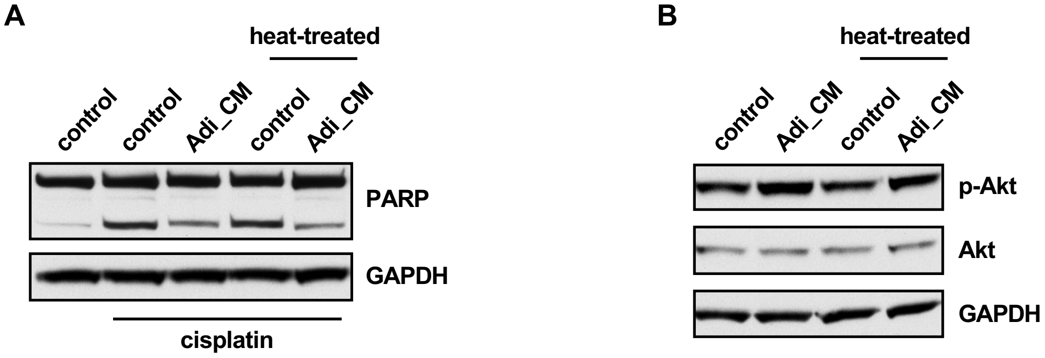

Supplemental Figure 4. The secreted factor(s) from adipocytes that promotes chemoresistance is heat-resistant. Heat treatment of adipocyte CM did not alter the capability of the CM to inhibit cisplatin-induced apoptosis (**A**) or to induce Akt activation (**B**).

Supplemental Figure 5

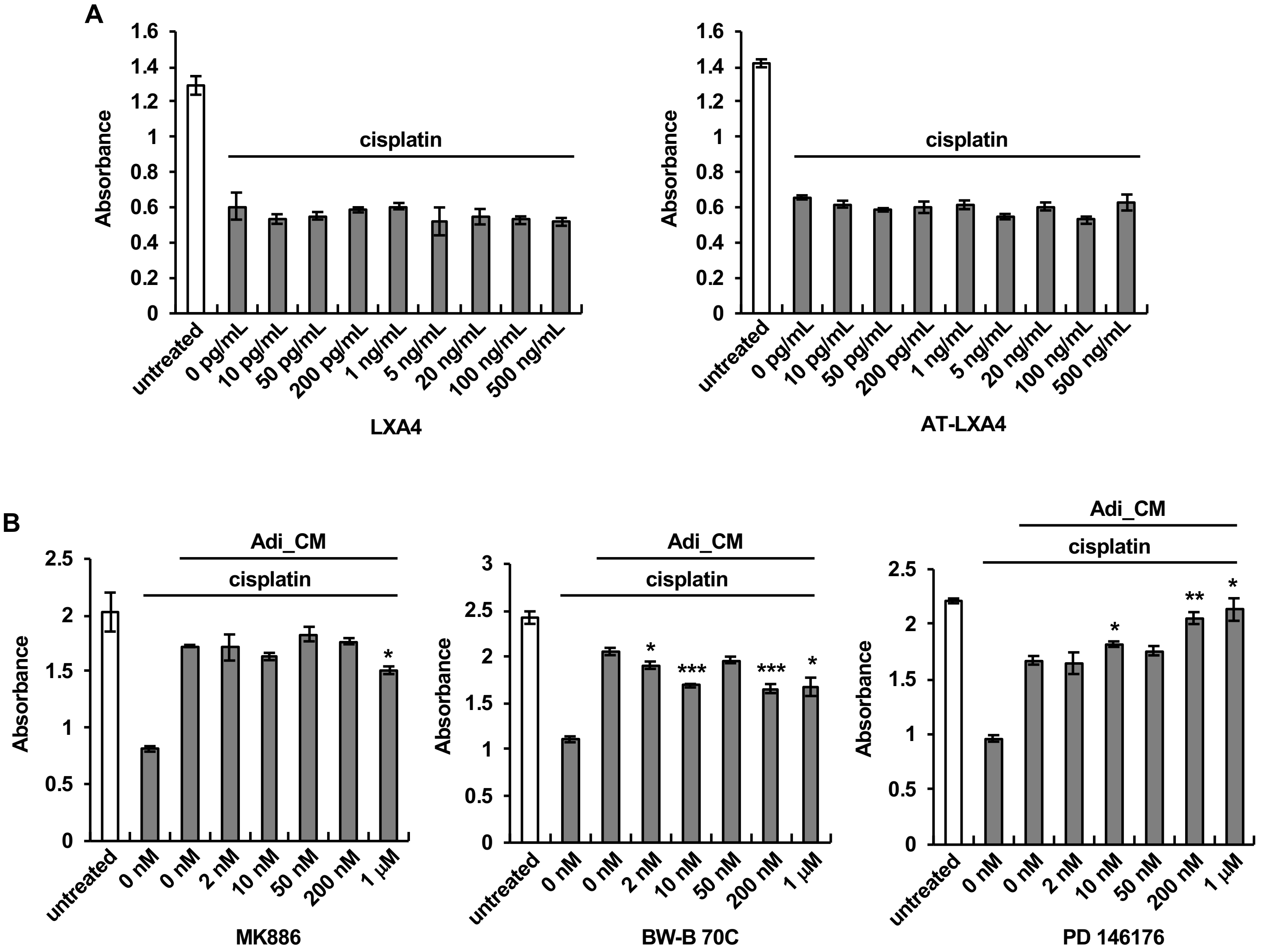

Supplemental Figure 5. Lipoxins do not mediate adipocyte-induced chemoresistance. OVCAR5 cells were treated with cisplatin together with different concentrations of lipoxin A_4 (LXA_4) or AT-LXA_4 (**A**). Cell viability was analyzed 72 hours later. **B**) Adipocytes were treated with inhibitors against 5- or 15-LOX (MK886, BW-B 70C and PD 146176) before CM was collected. CM deficient of lipoxins was then applied on ovarian cancer cells together with cisplatin. * p<0.05, ** p<0.01 and *** p<0.001, as compared to cells treated with cisplatin and control Adi_CM.

Supplemental Figure 6

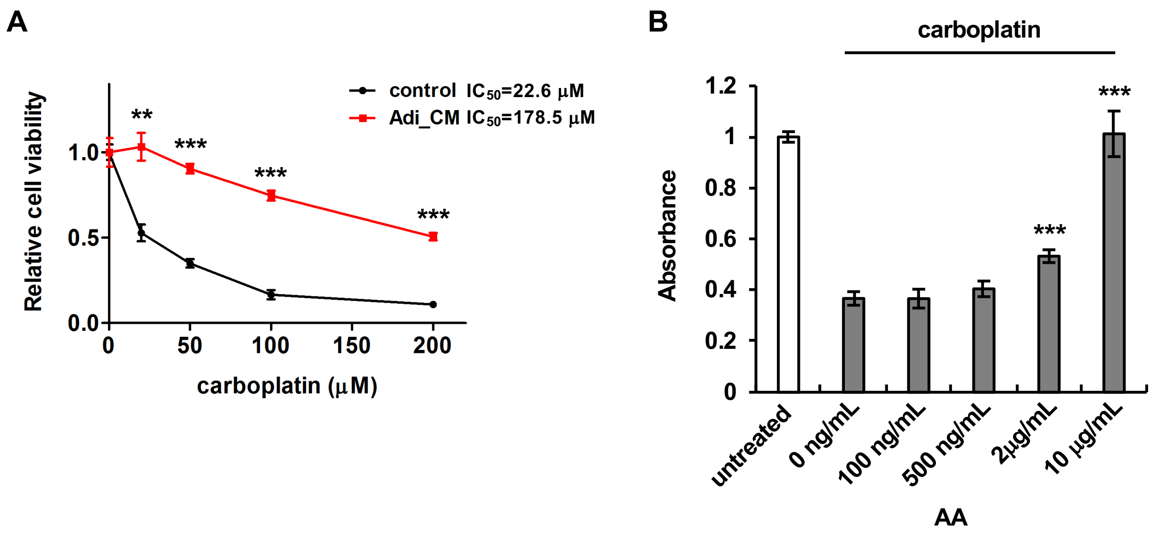

Supplemental Figure 6. Adipocyte CM and arachidonic acid increase ovarian cancer cell chemoresistance to carboplatin. A) OVCAR5 cells were incubated with adipocyte CM and different concentrations of carboplatin. **B**) OVCAR5 cells were treated with 50 μM carboplatin and different concentrations of arachidonic acid (AA). Cell viability was analyzed 72 hours later. ** $p<0.01$, *** $p<0.001$.

Supplemental Figure 7

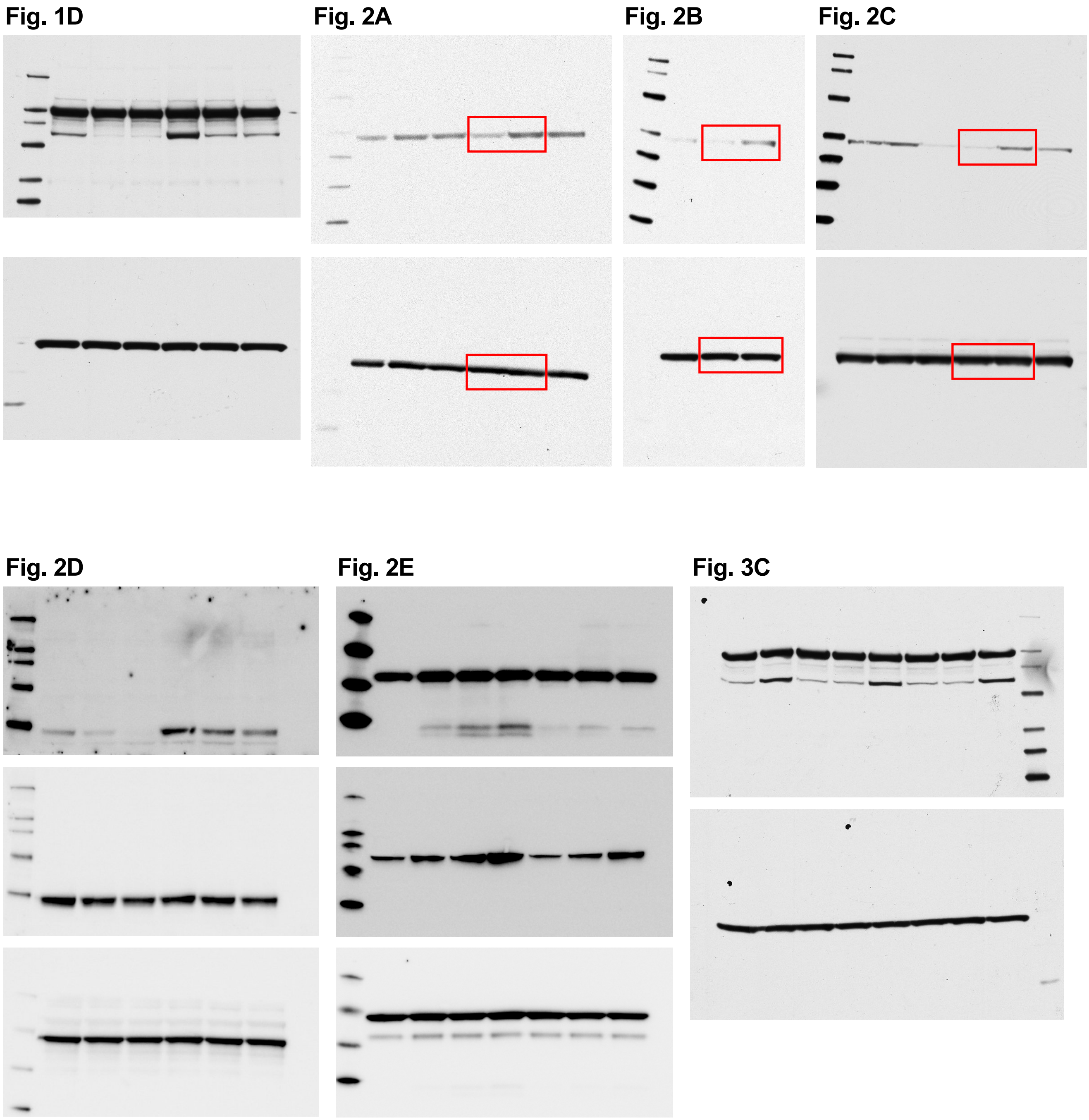

Supplemental Figure 7. Uncropped images of Western Blots from Figure 1-3.

Supplemental Figure 8

Fig. 4A

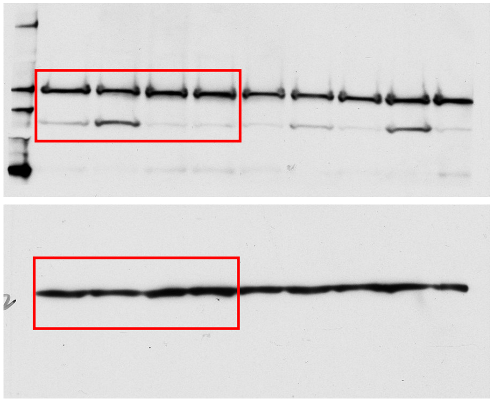

Fig. 5E

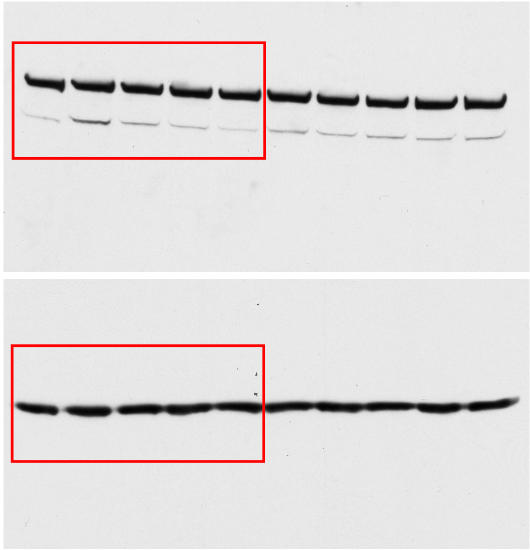

Fig. 5F

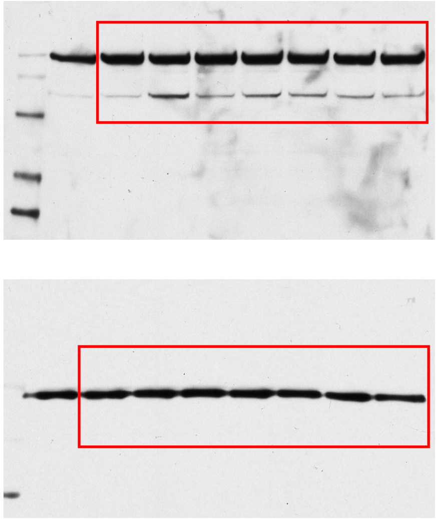

Fig. 6B

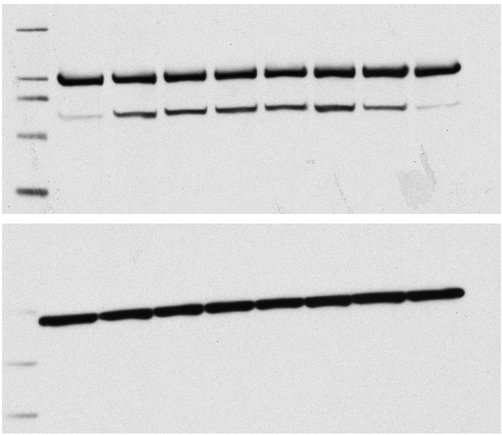

Fig. 6C

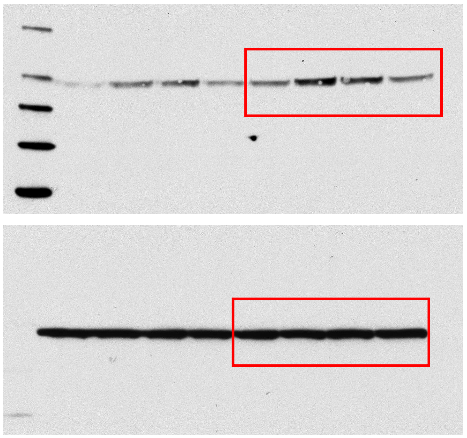

Supplemental Figure 8. Uncropped images of Western Blots from Figure 4-6.
